# Access without published appraisal: orphan medicine reimbursement and health equity in Türkiye

**DOI:** 10.1093/eurpub/ckag204

**Published:** 2026-11-04

**Authors:** Ferit Sevim, Çağdaş Erkan Akyürek

**Affiliations:** Department of Healthcare Management, Faculty of Health Sciences, Karadeniz Technical University, Trabzon, Türkiye; Department of Healthcare Management, Graduate School of Health Sciences, Ankara University, Ankara, Türkiye; Department of Healthcare Management, Graduate School of Health Sciences, Ankara University, Ankara, Türkiye; Department of Healthcare Management, Faculty of Health Sciences, Ankara University, Ankara, Türkiye

## Abstract

Rare diseases are individually uncommon but collectively affect a substantial share of any population. The medicines developed for them combine severe conditions, very small treatable populations, high prices, and evidence that is often immature at launch. Conventional cost-effectiveness analysis handles this poorly, and systems relying on it heavily tend to reimburse fewer orphan medicines than those applying alternative evidence standards. Türkiye offers an instructive current case. Of the 66 orphan medicines granted central marketing authorization in the European Union between 2021 and 2024, 3 were available in Türkiye at the start of 2026, against a European Union average of 28. The comparable figure for all new medicines was 9 of 168, against an average of 76. Restricted access to orphan medicines should therefore not be read solely as an orphan-specific exclusion, but as part of a broader constraint on new medicines, distinguished by one feature: a parallel route in which medicines are authorized for named patients, imported, and reimbursed outside the standard list. That route carries real access that list-based indicators capture only in part, and it makes a patient entitlement depend on which list a medicine occupies. A rule issued in June 2026 restricting reimbursement of spinal muscular atrophy medicines after gene replacement therapy illustrates the limited public availability of the clinical and economic reasoning behind reimbursement restrictions. Broadening the basis of appraisal, rather than reducing reliance on economic evaluation, requires published criteria, instruments for managing uncertainty, and monitoring of the pathway as well as the list.

## Background and public health problem

Rare diseases, defined in the European Union as those affecting fewer than five people per 10 000, are individually uncommon but collectively affect millions of Europeans [1]. The medicines developed for them combine small trials, often single-armed and reliant on surrogate endpoints, high prices, and evidence at launch that is frequently insufficient to estimate long-term outcomes [2].

High prices divided by modest or uncertain quality-adjusted life-year gains produce ratios far above conventional benchmarks, whatever the severity of the condition. Comparative work across 35 countries found that systems requiring formal clinical and economic evaluation reimburse a smaller proportion of orphan medicines than systems applying alternative evidence standards [3]. Gene and cell therapies sharpen the problem: the entire cost falls at a single moment while the durability of benefit remains unestablished.

The dilemma is a public health one: a payer applying a single efficiency rule will exclude a small, severely affected population, while a payer suspending it commits large sums to treatments of unproven durable benefit. Türkiye illustrates this tension. Consanguineous marriage, one contributor to rare disease risk, is a notable feature of the context [4]; rare disease care is a stated ministerial priority [5]; and researchers and clinicians report persistent diagnostic delay and uneven specialist capacity [6]. Statutory insurance covers almost the whole population, so the question is not whether public funds will be committed but on what basis, through which channel, and with what consistency.

## Context

The Social Security Institution (SGK) is the single public purchaser of health care in Türkiye. Reimbursement operates through a positive list: a company applies after marketing authorization, a Medical and Economic Evaluation Commission reviews a dossier including a pharmacoeconomic evaluation, a reimbursement commission decides, and listed products appear in Annex 4/A of the Health Implementation Communiqué [7, 8].

Türkiye has not established an independent health technology assessment (HTA) body; elements of HTA are applied within government institutions [7, 9], and no published threshold or appraisal methodology governs reimbursement decisions. Nor is there an orphan-specific appraisal pathway. Türkiye applies the European prevalence threshold to define a rare disease and defines an orphan medicine in its 2007 pricing communiqué, but neither establishes designation, incentives or a distinct appraisal route [10]. Draft national guidance finalized in 2016 was never issued; an end-2018 target of an orphan medicine policy was not met; and a ministerial department followed in 2020, alongside a parliamentary commission calling for specialized centres and registries [10, 11].

A parallel route has long carried much of the burden. Since a 2007 protocol between the payer and the pharmacists association, medicines unlicensed or unavailable domestically may be authorized for named patients, imported, and reimbursed if listed in Annex 4/C, on grounds of public health need [7, 8, 10]. A systematic review of Turkish pharmacy policy research concludes that this route is inadequate for orphan medicines under current pricing and reimbursement conditions [12]. An analysis covering 2017 put its cost at around US$520 million, 7.5% of public pharmaceutical expenditure, with the 10 most expensive products, 9 of them orphan medicines, accounting for about US$408 million and 7000 patients [7]. No more recent analysis of comparable scope was identified. Two instruments followed: the Alternative Reimbursement Regulation of 2016 [13], and Law No. 7151 of 2018, time-limiting a product presence on that list unless marketing authorization is pursued [7, 14]. A marketing authorization regulation of December 2021, aligned with European Union legislation, introduced conditional and exceptional-circumstances authorization: a product may be licensed on incomplete data for 1 year at a time, subject to published obligations and periodic benefit-risk review [15].

## Description of the initiative, policy, or intervention

The case examined here is how that architecture behaved during 2025 and 2026, when Türkiye extended coverage for several rare conditions and, in the same period, began restricting it by rule. The documents examined, retrieved from the Official Gazette and the payer’s published annexes and current to September 2026, were the Communiqué and its annexes in the versions in force, the amending communiqués of 2025 and 2026, the regulations and laws cited, and the payer’s annual activity report. The review focused on provisions concerning reimbursement criteria, consultation, and reassessment. Annex entries were counted at product level from the published files and de-duplicated by barcode. Four elements make up the initiative.

### List expansion

The institution reports 8474 medicines on the domestic reimbursement list at the end of 2025, with 455 added that year against 425 in 2024 and 346 in 2022 [16]. These totals cover the whole benefit package and do not themselves measure rare disease coverage. Within them, additions included subcutaneous prophylaxis in haemophilia A (March 2025), an oral option in spinal muscular atrophy (May 2025) and an oral alternative to enzyme replacement in Fabry disease (January 2026), while combination therapy for cystic fibrosis remains reachable only through the foreign list [8].

### A categorical restriction

The amendment published on 29 June 2026 provides that, because gene replacement therapy is administered once, the cost of nusinersen or risdiplam used after it is not met by the institution [17]. None of the annexes examined contains onasemnogene abeparvovec, whereas nusinersen appears on the domestic list, the foreign medicines list, and the list exempt from co-payment [8]. The import route, meanwhile, is being maintained rather than wound down: additions to a monitoring system for that list are a formal performance indicator [16].

### Analytic capacity building

In February 2026, the institution signed a protocol on health policy and financing with the national health institutes and two universities [16].

### A planning layer

The Rare Diseases Health Strategy Document and Action Plan 2023–27 spans awareness, screening, diagnosis, treatment, and research [5] but does not set out a value assessment methodology. Together, these developments show a payer extending coverage for some rare disease treatments, introducing a rule-based reimbursement restriction, and investing in analytic capacity during the same period.

## Implementation process

Implementation runs through amendment of the Health Implementation Communiqué, revised in 7 separate months of 2026 alone, each time altering annex lists, prescribing conditions, or payment rules across the benefit package [8, 17]. The medicines agency authorizes individual use, the pharmacists association imports and distributes, and SGK commissions set coverage and price [7, 8].

The amendments cited here are enacted as regulatory text, and none of those examined was accompanied by a published appraisal report, economic evaluation, or statement of reasons. No formal public consultation stage and no scheduled reassessment tied to outcome data were identified in the documents reviewed, although the absence of a published procedure does not establish that decisions are never revisited. The machinery is fast and legally clear, but it leaves little public record of the reasoning behind a decision.

## Observed outcomes or early signals

Few newly authorized orphan medicines reach patients in Türkiye through the standard route. Of the 66 granted central authorization in the European Union between 2021 and 2024, three were available in Türkiye at 5 January 2026, against a European Union average of 28; one surveyed country recorded fewer. The survey counts a medicine as available once it enters the public reimbursement list; all three qualified on that basis, without the restrictions recorded for 38% of available orphan products across the European Union [18].

That figure should be read alongside the all-product figure: 9 of 168, against an average of 76 [18]. Orphan medicines are thus not distinctly excluded relative to other new medicines; both sit far below European averages. What distinguishes the orphan case is that parallel route. The foreign medicines list holds 413 entries covering 246 active substances, more than half priced below 100 euro and including ordinary products such as warfarin and colchicine. Its upper tail differs: nusinersen, at a listed price of 73 000 euro per vial, is the highest-priced entry and appears on both annexes, though listed prices do not show net cost [8]. A channel built for supply gaps also carries the costliest rare disease treatments.

The same survey records a median time to availability of 435 days in Türkiye against a European average of 525. The apparent advantage is an artefact: the interval runs from national rather than European authorization and rests on three products. The survey tracks a defined cohort and records individual-patient access separately; within that scope, it is sound. It should not be interpreted as a measure of overall access in a system where much rare disease treatment predates the cohort and is funded through importation.

The amendment gives a stated rationale, that gene replacement therapy is administered once, but does not publish the clinical or economic assessment behind it. Uncertainty about the durability of that therapy does not by itself establish the value of subsequent treatment either. The defensible concern is narrower: a restriction with material consequences for an identified group rests on reasoning unavailable for scrutiny. No published analysis of how many patients it affects was identified.

## Challenges and unintended consequences

Budget rules in place of published appraisal. Declining to fund two therapies aimed at the same objective is defensible; the difficulty is the form the decision takes. One structured response would be to treat durability as uncertain and to use conditional, time-limited coverage tied to registry follow-up and reassessment, as European expert groups and modified national pathways have proposed [2, 19]. A categorical exclusion establishes a clear reimbursement rule, but the absence of a published assessment makes it difficult for external stakeholders to evaluate how the relevant clinical and economic considerations informed that rule.

### Contestation without a forum

The rules cited here provide little publicly documented rationale for clinicians, patient organizations or researchers to scrutinize, and no procedure for revisiting a contested judgement was identified. The publicly available documents do not establish whether a broader value framework informs these decisions.

### A tiered entitlement

The channel through which a medicine is funded determines the character of the claim. Annex 4/A listing creates a rule-based entitlement; Annex 4/C creates one contingent on per-patient authorization, and therefore on clinician familiarity and on family capacity to sustain repeated administrative steps. In a national survey, 73% of rare disease patients and families identified reaching treatments as a principal obstacle, around 30% of carers had left paid work and 25% of patients aged 0–18 had left school [11]. These are reported difficulties from a self-selected sample predating the arrangements examined here; they indicate the kinds of barrier patients encounter, not the effect of these rules. Non-listing leaves out-of-pocket payment. The June 2026 rule adds a further distinction, defining a group of patients by a therapy they previously received. Because no reimbursement annex lists that therapy, the patients it identifies are likely to be those whose families obtained it outside the scheme. Prior gene replacement therapy may be clinically relevant to the decision; the difficulty is that the published reasoning does not explain why it should remove entitlement to the subsequent medicines.

### Geography and specialist capacity

Consanguineous marriage, one contributor to rare disease risk, varies between provinces from 0.4% to 16.9% of registered marriages [4]; the distribution of specialists is uneven [6]; and 81% of patients and families nationally identified reaching an appropriately specialized physician as a principal obstacle [11]. Where access begins with an individual clinician application, these conditions may influence who is able to navigate the process, and so who obtains coverage.

### Uncertainty negotiated rather than managed

The Alternative Reimbursement Regulation covers rare disease and high-budget medicines and permits performance-based as well as financial models; the institution reports signing such agreements during 2025 for haemophilia A, a rare disease biosimilar and spinal muscular atrophy [13, 16]. Terms and outcomes are not publicly available [7]. Conditional authorization has existed on the licensing side since 2021, with time-limited validity, mandatory evidence generation and scheduled reassessment. No counterpart in reimbursement was identified, and advanced therapy medicinal products, the class to which gene replacement therapy belongs, fall outside its scope [15]. Expenditure concentration in a route without published appraisal is a legitimate concern for budget predictability, though listed prices and historical figures cannot establish current financial vulnerability. The absence of a dedicated HTA body may constrain reassessment capacity; the institution reports assessment output in single figures a year [9, 16]

## Lessons for public health

Administrative access is not the same as equitable entitlement. Türkiye illustrates how a single-payer system can provide access to high-cost orphan medicines through exceptional channels, although the current scale of access through these routes remains unclear. It also suggests that access delivered this way may be shaped by route, geography and administrative navigation as well as need.

Broaden the basis of appraisal rather than abandon economic evaluation. Conventional cost-effectiveness criteria cannot carry the full weight of orphan medicine decisions, but setting appraisal aside does not solve the problem. Where no appraisal framework or decision rationale is publicly available, external observers cannot determine how clinical need, uncertainty, cost-effectiveness, and budget impact were weighed in individual reimbursement decisions. Publishing the criteria and reasoning would make these decisions more transparent and open to scrutiny. A multi-criteria decision analysis framework is one possible approach to making competing considerations explicit, particularly where the criteria and their weights are publicly reported [2, 19].

Extend existing instruments rather than import new ones. Türkiye has already built, in its licensing regulations, machinery for acting under uncertainty: 1-year authorizations, published obligations, mandatory studies, and periodic benefit-risk review [15]. Applying that logic to coverage requires no new principle, though extending it to the payer and to product classes outside its scope is not a small undertaking. Conditional coverage depends on registries that are governed and maintained, on follow-up after listing, and on the capacity to act when reassessment points towards withdrawal or renegotiation. Publishing criteria is the cheapest step; sustaining the machinery is not. Measure the pathway, not the list. Cross-country indicators count admission to public reimbursement lists, as intended; they may not capture the full extent of access in systems where named-patient import routes are also used. Monitoring should record who obtains a medicine, through which route, after what delay and with what out-of-pocket exposure. The gap between what an indicator counts and what a system funds is in the first instance a measurement problem; it becomes an equity question when the uncounted route carries different obligations for patients, as it does here.

## Transferability and limitations

The case is transferable to systems with a dominant public purchaser, external reference pricing, a named-patient import channel, and no dedicated HTA institution. The sequence suggested here is to publish appraisal criteria before building the institutions to apply them, though drafting criteria that command assent is itself demanding. European accession adds a consideration: joint clinical assessment extends to orphan-designated medicines from 2028 [20], supplying a shared input to national appraisal.

Several limitations apply. The central events are recent and unevaluated; this is an early policy analysis, not an impact assessment. The documentary review was targeted rather than systematic, and later amendments may alter the arrangements described. The cross-country data are compiled from industry associations, and the annex analysis counts entries, not molecules. Confidentiality of managed-entry terms precludes independent assessment of value for money, and patient-level access data by region or income are not public. The argument concerns documented arrangements and the mechanisms they plausibly create, not demonstrated effects on patients. Türkiye is not representative of its region; the tension between access, uncertainty, and affordability is simply visible there with particular clarity.

## Data Availability

The source documents and published cross-country availability data analysed in this article are publicly available. The extracted dataset and counting script are available from the corresponding author on reasonable request.
